# New insights into the regulation of synaptic transmission and plasticity by the endoplasmic reticulum and its membrane contacts

**DOI:** 10.2183/pjab.97.028

**Published:** 2021-12-10

**Authors:** Masafumi TSUBOI, Yusuke HIRABAYASHI

**Affiliations:** *1 Graduate School of Engineering, The University of Tokyo, Tokyo, Japan.

**Keywords:** endoplasmic reticulum, neurotransmission, synaptic plasticity, ER-mitochondria contact, serial scanning electron microscopy

## Abstract

Mammalian neurons are highly compartmentalized yet very large cells. To provide each compartment with its distinct properties, metabolic homeostasis and molecular composition need to be precisely coordinated in a compartment-specific manner. Despite the importance of the endoplasmic reticulum (ER) as a platform for various biochemical reactions, such as protein synthesis, protein trafficking, and intracellular calcium control, the contribution of the ER to neuronal compartment-specific functions and plasticity remains elusive. Recent advances in the development of live imaging and serial scanning electron microscopy (sSEM) analysis have revealed that the neuronal ER is a highly dynamic organelle with compartment-specific structures. sSEM studies also revealed that the ER forms contacts with other membranes, such as the mitochondria and plasma membrane, although little is known about the functions of these ER-membrane contacts. In this review, we discuss the mechanisms and physiological roles of the ER structure and ER-mitochondria contacts in synaptic transmission and plasticity, thereby highlighting a potential link between organelle ultrastructure and neuronal functions.

## 1 Introduction

Unlike most cell types in the mammalian system, the majority of neurons in the central nervous system are produced during development and have to be maintained throughout the lifetime of an organism. Therefore, neuronal organelles and proteins are actively replaced to keep neurons functional for decades. Indeed, numerous genetic mutations in genes regulating organelles, including the endoplasmic reticulum (ER) and mitochondria, are linked to neurodegenerative diseases. The ER serves as a platform for important biochemical reactions, such as protein and lipid synthesis^1,2)^ and Ca^2+^ release.^3)^ Besides such functions, the ER also monitors normal protein folding, trafficking, and degradation. This checkpoint by the ER is well known as ER stress. Disturbance of this system leads to the accumulation of pathological protein aggregates and neurodegenerative diseases.^4)^ Although neurons have multiple functionally compartmentalized domains, a cell body, dendrites, and an axon, the ER is composed of a continuous lipid bilayer throughout a cell.^5–9)^ Similar to other cell types, the ER surrounds the nucleus and forms the nuclear envelope (NE) in the cell body of a neuron. Cisternae and tubular forms of the ER branch out from NE to form the peripheral ER, which extends throughout the neuron. Previous studies revealed that the ER contributes to the regulation of synaptic transmission and plasticity in the dendrite^10–17)^ and the axon.^18–25)^ Recently, the rapid development of serial scanning electron microscopy (sSEM) analyses have revealed membrane-membrane contacts between the ER and other organelles in neurons.^26,27)^ Among such contacts, mitochondria-ER contacts (MERCs) are the most frequently observed in many cell types and are known to represent signaling hubs for various biochemical reactions. For example, mitochondria uptake Ca^2+^ released from the ER through a mitochondria Ca^2+^ uniporter (MCU) complex at this contact site. Furthermore, lipid exchange between the ER and mitochondria is required for the synthesis of glycerophospholipids, which are the major components of the plasma membrane.^28–30)^ Additionally, MERCs have been suggested to define division sites of mitochondria,^31–33)^ thereby regulating mitochondrial dynamics. Such functions of MERCs provide a new insight that the ER does not function as an isolated unit, but rather communicates with other organelles and forms dynamic membrane contacts in response to cellular demands in many cell types including neurons. Thus, studying the roles of these contacts in neurons holds promise for revealing new insights in the cellular mechanisms of synaptic transmission. Here, we review our current understanding of ER functions in neurons and novel modalities of ER-(other) membrane contacts in the regulation of neurotransmission.

## 2 Structural features and dynamics of the ER in axons

**Figure 1.**
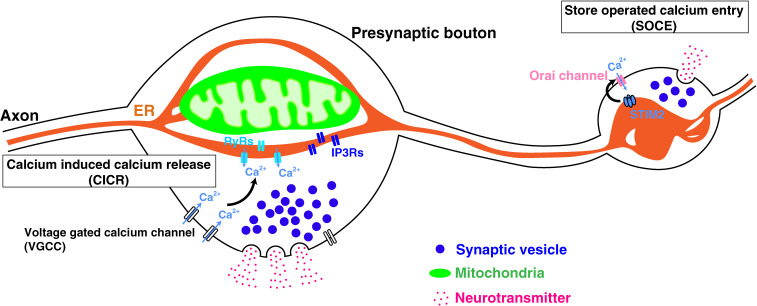
(Color online) Structural features and functions of the axonal endoplasmic reticulum (ER). The ER represents thin and tubular morphology in the axon shaft, while it bulges specifically at the presynaptic site in the cortex and nucleus accumbens.^26)^ The ER at presynaptic varicosities take a variety of different forms including a simple sheet or branched small cisternae.^26,36)^ The ER contributes to the neurotransmission and plasticity through the regulation of presynaptic Ca^2+^ homeostasis.

The axonal ER is continuous from the cell body and runs parallel to the axonal shaft as tubular structures with a similar diameter to the tubular ER in the cell body.^9,34,35)^ In addition, a web of anastomosed tubules made of smooth ER (SER) is present both myelinated and unmyelinated large axons of spinal ganglion neurons.^9)^ Recently, advances in the automated serial electron microscopy (EM) have provided high-resolution and large-volume ultrastructural three-dimensional analyses of the axon in the brain. These studies showed that in the cortex of mice the thinnest (∼200 nm in diameter) unmyelinated axons usually contained a single narrow ER tubule, consistent with previous reports.^26,36)^ At the branching points of SER tubules, they did not connect with each other directly but rather formed a comparably small lumen called cisternae.^36)^ Notably, the structures of the ER in presynaptic varicosities take a variety of different forms.^26,27)^ In the nucleus accumbens, the presynaptic ER forms branches and expands into small cisternae.^26)^ In addition, in some presynaptic sites, ER-like tubules physically discontinuous from the ER network have been observed. On the other hand, in pyramidal neurons in layer 4/5 of the mouse neocortex, the ER bulges specifically at presynaptic sites and shows a simple sheet-like structure.^27)^ Even though there are structural differences in the presynaptic ER, both reports showed that the ER forms extensive contacts with mitochondria in presynaptic varicosities, confirming studies using conventional serial EM techniques^37,38)^ (Fig. 1).

The interaction between the ER and cytoskeleton generates the main mechanical force for membrane deformation in shaping ER networks.^39)^ Studies in non-neuronal cells have suggested that the ER-microtubule (MT) interactions are required for the formation of elongated ER tubules.^40–44)^ Indeed, the ER binds to MTs in the axon.^42,45–47)^ Myosin Va (Myo-Va) is likewise involved in distributing axonal ER along an intermediate filament, Neurofilament-L.^48)^ In contrast to the cytoskeleton that functions throughout all neuronal compartments, ER-resident proteins have been shown to regulate ER morphogenesis particularly in the axon. Genetic mutations in the ER-resident proteins Reticulon (RTN)-2, REEP1, and atlastin-1 are associated with an axonopathy, hereditary spastic paraplegia,^49–51)^ and a mutation in the ER membrane receptor VAMP-associated protein (VAP) B is associated with another type of axonopathy, amyotrophic lateral sclerosis.^52)^ In Drosophila, deletion of Reticulon-like-1, the Drosophila ortholog of RTN, and Reep proteins causes a reduction in SER in the distal portion of motor neurons and occasional discontinuities in the axonal ER.^53,54)^ Ultrastructural analysis of the axonal ER in larvae lacking reticulon and Reep proteins showed that the diameter of the ER was increased and the number of ER tubules per section was reduced,^54)^ consistent with the molecular functions of hereditary spastic paraplegia-related proteins in stabilizing ER membrane curvature.^39,55)^ Lindhout *et al.* showed that VAPB and its family protein VAPA interact with cytoplasmic protein secernin-1 (SCRN1) at the ER membrane via a single FFAT-like motif, and this interaction regulates ER morphology in the axon.^56)^ Notably, loss of VAP–SCRN1 interactions resulted in a decrease in synaptic vesicle cycling. Consistent with this finding, VAP or SCRN1 knockdown both resulted in a decrease in action potential (AP)-evoked presynaptic Ca^2+^ influx, suggesting that VAP–SCRN1-mediated ER remodeling regulates presynaptic Ca^2+^ homeostasis and thereby modulates AP-evoked synaptic transmission.

In addition to the regulation of ER morphology, a recent study showed that the amount of ER in the axon shaft and the presynaptic bouton is regulated by ER membrane turnover via autophagy (ER-phagy).^57)^ Deletion of ATG5, an essential protein for the autophagosome formation, leads to the accumulation of SER proteins, particularly tubular ER forming proteins, such as RTN3 and VAPB. Furthermore, ultrastructural analysis showed elevated numbers of ER tubules specifically in axons and at presynaptic sites. This indicates that neuronal autophagy constantly degrades SER in the axon. Moreover, ATG5 knockout caused an elevation of ryanodine receptor (RyRs)-mediated Ca^2+^ release from the ER, resulting in an increase in spontaneous release.^57)^

Although live imaging of the axonal ER labeled with GFP-fused CG9186, an ER localized lipase, showed anterograde and retrograde movements of ER tubule-like structures detached from the ER networks in Drosophila neurons,^54)^ when and how such dynamic movements of the axonal ER occur remain unclear.

## 3 ER functions in the axon

Mature axons exclusively exhibit smooth ER and are devoid of rough ER.^35,37,53,58,59)^ This indicates that the axonal ER is likely to have functions other than protein synthesis. Although Ca^2+^ import from the extracellular space mediated by voltage-gated Ca^2+^ channel has been mainly focused on in terms of presynaptic Ca^2+^ regulation, it has been revealed that the SER functions as a Ca^2+^ source in the axon in several types of neurons.^18–20,23)^ The increase in cytoplasmic Ca^2+^ can cause calcium-induced calcium release (CICR) mediated by RyRs localized in the ER membrane. CICR was shown to be required for the induction of spontaneous but not evoked neurotransmitter release by single APs with low-frequency (∼0.1 Hz) stimulation.^18–22)^ In addition to the functions in the induction of spontaneous neurotransmitter release, RyRs were shown to be required for the enhancement of evoked GABAergic synaptic currents at the cerebellar basket cell**–**Purkinje cell synapse.^24)^ Furthermore, although the role of CICR in the induction of short-term plasticity remains controversial,^20,21)^ presynaptic CICR was shown to be required for inducing long-term depression (LTD) in hippocampal pyramidal neurons.^22,23)^ Notably, CICR-mediated spontaneous firing is depotentiated by ER-phagy-mediated reduction in the amount of ER.^57)^ In addition to CICR, presynaptic enrichment of inositol trisphosphate receptor (IP3R) 1 was observed in presynaptic terminals of the hippocampal excitatory Schaffer collateral synapses upon tetanic stimulation.^60)^ Furthermore, IP3R-induced Ca^2+^ release was shown to induce GABA release downstream of kainate receptors in rat prefrontal GABAergic neurons.^61)^ Consistent with these reports, impairment of ER structures by loss of ER-resident proteins such as atlastin, reticulon, and VAPs, is accompanied by a decrease in neurotransmitter release.^56,62,63)^ These reports suggest that the ER functions as an accelerator of presynaptic release probability. In correlation with presynaptic Ca^2+^ influx, net uptake of Ca^2+^ was induced by neuronal activity through Sarco/ER Ca^2+^-ATPase.^64)^ Further studies are required to resolve the complex interplay among different sources of Ca^2+^ at presynaptic boutons. In addition to the functions of the ER itself as a Ca^2+^ source or sink, store-operated calcium entry (SOCE) activated by ER Ca^2+^ depletion is known to increase the frequency of spontaneous neurotransmitter release in hippocampal neurons.^20,65,66)^ In many cell types, Ca^2+^ concentration in the ER lumen is sensed by ER-resident stromal interaction molecule (STIM) family proteins. Two isoforms, STIM1 and STIM2, are known to be expressed in neurons and both proteins have weak affinity to Ca^2+^ via an EF-hand motif at the N-terminus. When luminal Ca^2+^ concentration in the ER decreases, the unoccupied EF-hand motif induces a conformational change of the STIM protein, leading to its clustering and activation. This in turn activates Orai, a plasma membrane Ca^2+^ channel, to induce Ca^2+^ influx. Of note, depleting Ca^2+^ from the ER increases miniature excitatory postsynaptic current (mEPSC) frequency through a mechanism requiring STIM2 but not STIM1. This suggests that STIM2-mediated SOCE potentiates spontaneous neurotransmitter release. Given that SOCE is associated with several neurological disorders such as Huntington’s, Parkinson’s, and Alzheimer’s diseases (AD), it is possible that the aberrant control of SOCE-mediated spontaneous firing leads to the onset of such neurological disorders.

## 4 Structural features and dynamics of the ER in the dendrites

### 4.1 Structural features and dynamics of the ER in the dendritic shafts.

In dendrites, a large portion of the ER is SER elongated from the peripheral ER in the cortex of the cytoplasm as a continuous anastomosing network.^8,67)^ Serial EM and fluorescence recovery after photobleaching (FRAP) assays showed that the ER in aspiny segments of the dendrites consists primarily of multiple tubules along the shafts with thin branches intermittently traversing the cytoplasm. At branch points and the dendritic shafts near the spines, the ER becomes cisternae and is enriched with ribosomes.^8,68)^

**Figure 2.**
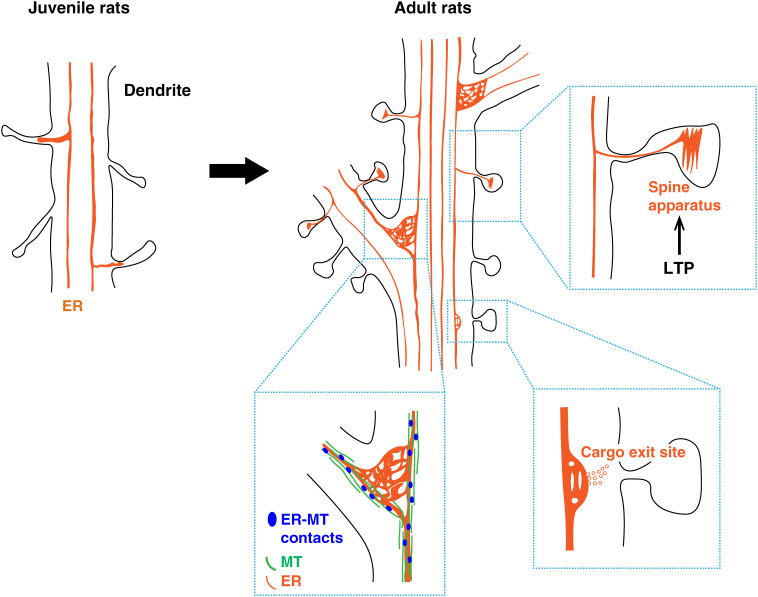
(Color online) Structural features and functions of the dendritic endoplasmic reticulum (ER). In juvenile rats, the dendritic ER consists primarily of multiple tubules along the dendritic shaft and its volume increases near dendritic spines in hippocampal pyramidal neurons. In adult rats, the ER shows more heterogenous and complicated structures with branched tubules and larger convoluted ER membranes (cisternae) at the branch point of dendrites and the shaft near the spines.^8,67)^ Elongated ER tubules in the dendritic shaft frequently interact with microtubules, whereas cisternae at the branch point are loosely associated with microtubules. These local cisternae serve as spatially compartmentalized cargo exit sites in the ER.^8)^ Smooth ER (SER) in mushroom spines or enlarged spines upon long-term potentiation (LTP) induction often shows folded or cistern structure called a spine apparatus (SA).^67,68)^ The SA is suggested to be important for LTP induction by serving as a Ca^2+^ store. Images have been adapted and modified with permission from Cui-Wang *et al.* (2012).^8)^

As in the axon, elongated ER tubules in the shaft frequently contact MTs in dendrites. On the other hand, at the branching points where the ER forms complex structures, the ER is much more loosely associated with MTs (Fig. 2).^8)^ The dendritic ER is associated with MTs via an ER integral membrane protein Climp63, which becomes enriched in the somato-dendritic compartment during neuronal maturation.^69)^ Notably, knockdown of Climp63 increased branches in the dendrites as well as ER complexity.^8)^ This indicates that ER complexity regulates dendritic branching. Mutual interactions between ER and MTs were also observed in *Caenorhabditis elegans* sensory PVD neurons.^70)^ It has been shown that ER extensions into the secondary and tertiary branches of PVD neurons are dependent on MTs. ER-associated MTs were more stable than the non-ER-associated MTs. These results indicated that the ER and MTs mutually affect each other, and the ER invades the dendritic branch through MT-dependent mechanisms.

Regulation of ER morphology by extrinsic cues is an intriguing process to study. For example, an extrinsic signal through group I metabotropic glutamate receptors (mGluRs) phosphorylates Climp63 via activation of protein kinase C and releases the ER tubules from MTs.^8)^ The source of the extrinsic cue and interplay with other signaling pathways remain to be elucidated.

### 4.2 Structural features and dynamics of the ER in the spine.

The contiguous ER in the dendritic shaft extends into the tips of spines. The insertion of the SER into spines appears to be differentially regulated among different types of neurons. Whereas most of the spines in Purkinje neuron are filled with SER,^71–73)^ less than 20% of spines contained SER in rat hippocampal CA1 dendrites.^67,68,74,75)^ A detailed three-dimensional analysis of the ER structure identified the SER and several types of non-SER compartments including clathrin-coated vesicles and pits, uncoated vesicles, tubules, and multivesicular bodies in dendritic spines of the rat hippocampal CA1 region.^67,68)^ The SER in large spines often shows a specialized structure called ‘spine apparatus’ (SA) (Fig. 2). This structure is characterized by folded or cisterns of SER and located in the spine neck or at the base of the spine head. The percentage of spines containing SAs was dramatically increased during the course of neuronal maturation,^67,68)^ whereas the percentage of spines containing SER was not significantly changed from postnatal day 15 to adulthood (Fig. 2). Given that mushroom-typed (perforated) large spines contain many more SAs in adult rats than those in younger rats,^67)^ it was suggested that the ER changes its structure along with spine maturation during development.

Studies using confocal microscopy showed that a majority of spine ER undergoes turnover in 24 hours but a small subset of spines maintain the ER for at least 4 days.^7)^ Recent time-lapse imaging of the ER using two-photon microscopy in an organotypic hippocampal slice culture showed that a significant fraction (about 70%) of spines were visited at least once during 5 hours of imaging.^76)^ These ER visits were typically short (more than 50% of visits were less than 10 minutes) and became significantly less frequent when excitatory transmission was blocked by the treatment with N-methyl-D-aspartate (NMDA) receptor inhibitor D-2-amino-5-phosphonovaleric acid (D-APⅤV) and α-amino-3-hydroxy-5-methyl-4-isoxazolepropionic acid (AMPA) receptor inhibitor NBQX. Notably, the ER preferentially visits spines enlarged by glutamate uncaging-mediated structural long-term potentiation (LTP).^76)^ These reports suggest that a significant fraction of spines is visited by the ER during excitatory transmission and LTP.

## 5 ER functions in the dendrites

What is the role of ER that is elongated in the dendritic shaft and is often inserted into the spine? It is a major challenge to supply lipids and proteins to dendritic spines located hundreds micrometer away from the cell body in response to fast-demand processes, such as synaptic potentiation. Classically, membrane proteins destined for the spine surface are thought to be exocytosed from the Golgi apparatus after being synthesized at the rough ER in the neuronal cell body. However, a recent study suggested that GluA, a core component of AMPA-type glutamate receptors (AMPARs), is transported to the spine via recycling endosomes from the dendritic ER in a Golgi apparatus-independent manner.^77)^ Additionally, cargo exit sites are enriched in dendritic shafts with more SER volume or branches^8)^ (Fig. 2). Considering the fundamental role of AMPARs in rapid excitatory synaptic transmission and plasticity, Golgi apparatus-independent transport from the SER located near the dendritic spine might facilitate the fast supply of membrane proteins and lipids to support synaptic potentiation. Consistent with this notion, the features of structural LTP, such as an increase in spine volume and surface area of the postsynaptic density (PSD), were observed preferentially in SER-containing spines. The reduction in the volume of shaft SER after LTP suggests that SER resources were utilized for spine outgrowth.^74,78,79)^ Together with the report that the density of SER-free spines was reduced upon LTP, this suggests that SER supplies protein- and lipid-containing cargoes to spines being enlarged during LTP.

Besides the role as an initiation site of endosomal trafficking, the SER serves as a site for efficient assembly of AMPARs. Schwenk *et al.*, performed proteomic analyses aiming for a comprehensive identification of proteins interacting with pore-forming components of AMPARs using membrane fractions from adult rat brains.^80,81)^ Notably, among identified novel subunits of AMPARs, FRRS1l is exclusively contained in an intermediate complex (priming complex) formed by the coordinated action of several proteins on the ER.^82,83)^ Moreover, disrupting the assembly of the intermediate complex by ablating FRRS1l significantly reduced the amount of AMPARs on the surface of the plasma membrane. This resulted in the impairment of synapse formation and the abolishment of LTP induction in hippocampal pyramidal neurons.^83)^ This report suggested that the SER functions as a platform for efficient AMPARs assembly and serves as a reserve pool of AMPARs for synaptic potentiation and transmission. Considering that the SA associates with large mushroom spines and large active zones, it is possible that the large amount of membrane provided by the cisternae structure of SAs is required for promoting the assembly of the priming AMPAR complex.

Then, the next question is how dynamic distribution of the ER is regulated in spines and what is the role of dynamic changes in the distribution of the ER during synaptic potentiation? Molecular motors moving along actin filaments play a critical role in bringing the ER network to the periphery of the neurons. Myo-Va is shown to be required for the insertion and elongation of the tubular SER from the dendrites to the spines in Purkinje neurons.^73)^ Notably, using Myo-Va-deficient Purkinje neurons, Miyata *et al.*, showed that SER insertion in the spine is required for the induction of LTD by increasing the local Ca^2+^ concentration.^11)^ Consistent with these reports in Purkinje cells, spines with SER are shown to be preferential sites for mGluR-dependent LTD in hippocampal pyramidal neurons.^75)^ These studies suggested that the insertion of SER into spines is required for proper synaptic plasticity. In line with these reports, Perez-Alvarez *et al.*, showed that expression of a dominant negative (DN) form of Myo-Va drastically reduced the frequency of ER visits into the spines and the number of spines with ER in hippocampal pyramidal neurons.^76)^ Expression of DN-Myo-Va significantly increased the spine surface expression of GluA2 and glutamate uncaging-evoked EPSCs in hippocampal CA1 pyramidal neurons. As a result, in these neurons, although low-frequency stimulation can induce LTD, pairing stimulation of both pre- and post-synapses did not induce LTP.^76)^ This suggested that transient visits of the ER maintain spines at intermediate strength for providing those spines with a potential for both LTP and LTD. Although a direct link between ER dynamics in the spine and the delivery of FRRS1l-containing AMPAR to synaptic surface membranes remains unclear,^76,83)^ both mechanisms may coordinately contribute to the induction of proper synaptic plasticity. It still remains unclear what molecules interconnect between the neuronal excitation and Myo-Va activity. The interplay of two Ca^2+^-binding proteins: calmodulin and caldendrin may fine-tune the activity of Myo-Va and thereby regulate the dynamics of the SER in spines in response to local Ca^2+^ increase.^84)^

In addition to the distribution of the ER in the spine, recent studies suggest that the ER changes its structural complexity in the spine after LTP. The ratio of spines containing SA to those containing a tubule SER was markedly increased 2 hours after theta-burst stimulation in hippocampal slice culture.^74,79)^ This suggested that a simple tubular SER in spines is transformed to a complex SA after LTP. The SA was shown to contain the actin-binding protein synaptopodin.^85)^ Synaptopodin-deficient neurons contained single ER tubules but completely lacked the SAs in the cortex, hippocampus, and striatum.^86)^ This absence of SA was accompanied by a decrease in LTP in hippocampal neurons, suggesting that this structure contributes to LTP.^86,87)^

## 6 ER-mitochondria contacts and their roles in mammalian cells

As mentioned above, the functions of the ER in dendrites have been rigorously studied. Although it has been suggested that part of the ER locates in close apposition to the mitochondria in dendrites from the early 1980s,^68,88)^ recent advances in sSEM techniques allowed us for the first time to investigate the contact between the ER and mitochondria comprehensively in 3D. This showed that there are extensive MERCs in all compartments of neurons.^27)^ However, the molecular mechanism underlying the formation of the ER-mitochondria contact in metazoans remained limited. Thus, the physiological significance of this contact had not been revealed.^89)^

Hirabayashi *et al.* recently identified the ER-resident protein PDZD8 as a critical ER-mitochondria tethering protein in mammalian cells including neurons.^90)^ In budding yeast, a tethering complex between the ER and mitochondria called ER-mitochondria encounter structure (ERMES) complex was identified in 2009.^89)^ Three out of four ERMES complex proteins (Mmm1, Mdm12, and Mdm34) contain a synaptotagmin-like mitochondrial lipid binding protein (SMP) domain.^91)^ However, no clear functional ortholog for any ERMES protein had been identified in metazoans. Recently, the crystal structure of an SMP domain was identified,^92–95)^ which allowed us to perform a more definitive structural homology search based on secondary and/or tertiary structures than that based on its primary amino acid sequence. This led to the identification of a candidate protein of unknown function containing a structurally-defined SMP domain, PDZD8. Indeed, a genetic complementation assay in budding yeast showed that the SMP domain of PDZD8 is functionally homologous to that of Mmm1.

Precise investigation of MERCs required EM due to the narrow (10–30 nm) distance between the ER and mitochondria at contact sites. In addition, both the ER and mitochondrial networks display highly complex topologies in 3D. Therefore, 3D rendering and quantification of MERCs areas have been very challenging in standard two-dimensional EM. To reveal the role of PDZD8 in the regulation of MERCs, Hirabayashi *et al.* used focused ion beam-serial EM, and reconstructed mitochondria and ER structures from several hundred serial EM sections. The extent of MERCs amount quantified from the 3D reconstruction revealed that the percentage of MERC area per mitochondria surface area was greatly reduced in PDZD8 knockdown HeLa cells compared with that in wild-type cells. This suggested that PDZD8 is required for MERCs formation.

**Figure 3.**
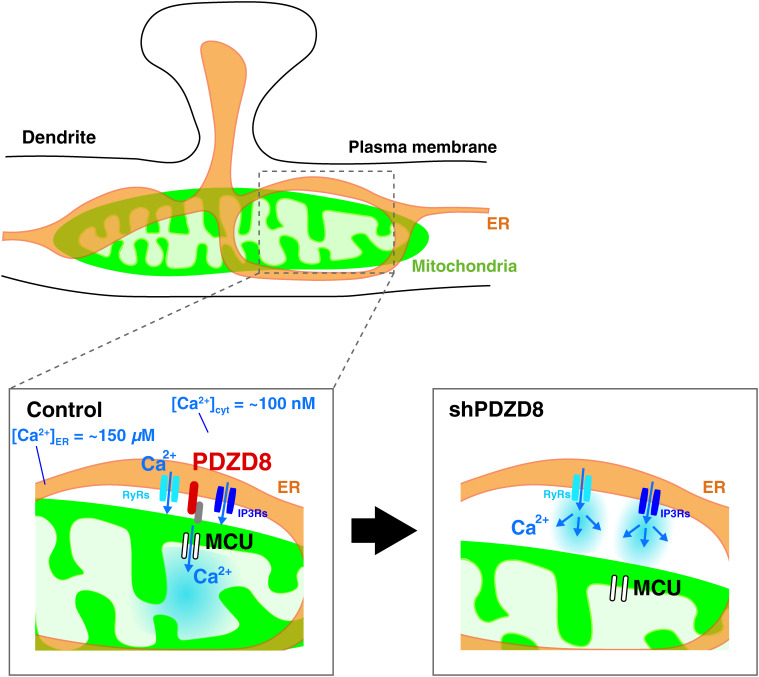
(Color online) The regulation of cytosolic Ca^2+^ homeostasis by endoplasmic reticulum (ER)-mitochondria contacts in the dendrite. Serial scanning electron microscopy (sSEM) techniques revealed extensive mitochondria-ER contacts (MERCs) in all the compartments of neurons. In dendrites of cortical pyramidal neurons, Hirabayashi *et al.*, showed that Ca^2+^ released from the ER upon synaptic stimulations is imported into dendritic mitochondria through mitochondrial calcium uniporter (MCU). In PDZD8-deficient neurons, a significantly higher fraction of Ca^2+^ released from the ER ends up in the cytoplasm rather than in the mitochondrial matrix. This suggests that PDZD8-dependent ER-mitochondria tethering plays a critical role in controlling synaptically induced elevation of cytoplasmic Ca^2+^. Images have been modified with permission from Hirabayashi *et al.* (2017).^90)^

As discussed above, the ER is an important source of cytoplasmic Ca^2+^. Therefore, whereas MERCs are proposed to serve as platforms for many important biochemical reactions, Ca^2+^ transfer from the ER to mitochondria is likely to be one of the main roles in neurons. The MCU, a Ca^2+^ channel in the mitochondrial inner membrane, is responsible for the mitochondrial uptake of Ca^2+^ released from the ER. To open the MCU, the concentration of Ca^2+^ on the mitochondrial surface must be greater than 1–5 µM depending on the components of the MCU complex.^96–98)^ However, the concentration of Ca^2+^ in the cytoplasm is usually less than 100 nM. It is only in the vicinity of the ER, that the cytoplasmic Ca^2+^ concentration reaches levels high enough to open the MCU. Thus, contact of mitochondria with the ER is required for mitochondrial Ca^2+^ uptake. It has been shown that the ER releases Ca^2+^ upon synaptic stimulation through IP3Rs and RyRs in the dendrite. This Ca^2+^ release can increase the local Ca^2+^ concentration of cytoplasm to a level higher than the threshold value for opening the MCU. Indeed, using high-speed dual-wavelength Ca^2+^ imaging and genetically encoded Ca^2+^ sensors specifically targeted to the ER or mitochondria, it was revealed that the ER releases Ca^2+^ upon electric stimulation and the mitochondria subsequently buffer Ca^2+^ in neocortical excitatory neurons *in vitro* (Fig. 3). Strikingly, PDZD8 knockdown decreased this mitochondrial Ca^2+^ buffering even though Ca^2+^ release from the ER was not significantly affected. This resulted in an increase in dendritic Ca^2+^ evoked by neuronal activity in the dendrites of PDZD8 knockdown neurons. This suggested that PDZD8-mediated ER-mitochondria tethering is essential for Ca^2+^ transfer from the ER to mitochondria and the regulation of cytoplasmic Ca^2+^. Given that Ca^2+^ released from the ER in the spine of Purkinje neurons is required for the induction of LTD,^11)^ MERCs may fine-tune it as another layer of synaptic plasticity. Additionally, physical tethering between the ER and mitochondria at MERCs can regulate transient visits of the ER into spines and may contribute to synaptic plasticity.^76)^

Considering that the ER wrapped around mitochondria in the presynaptic varicosities,^26,27)^ it is possible that the MERCs contribute to Ca^2+^ homeostasis also in the presynaptic bouton. Because Ca^2+^ uptake by presynaptic mitochondria^99,100)^ and presynaptic Ca^2+^ regulation by the ER have been observed during neuronal activity,^56,64)^ and how Ca^2+^ flux through MERCs, if any, contributes to presynaptic functions are of interest. Given that mitochondrial Ca^2+^ uptake activates the TCA cycle, such Ca^2+^ uptake via the MERCs may contribute to mitochondrial ATP synthesis to meet the energy demand during neurotransmission or synaptic plasticity. Further studies will reveal the physiological impact of the regulation of the ER-mitochondria contacts in the brain.

Abnormalities in MERCs have been reported in contexts related to various neurological diseases such as AD, Parkinson’s disease, and amyotrophic lateral sclerosis,^101–103)^ although it remains unclear how MERCs relate to the pathogenesis of these diseases. Considering that nearly half of sporadic AD patients exhibits hyperexcitability such as a epilepsy before the onset of symptoms,^104)^ aberrant formation of MERCs may be related to such symptoms in the early stage of AD through dysfunctions in Ca^2+^ homeostasis. Further studies investigating the functions of MERCs in neurons may provide new insights into the pathogenesis of neurodegenerative disorders as well as a basis for the development of new therapeutic strategies.

Studying the dynamics of MERCs in neurons will provide clues for revealing the link between neuronal activity patterns and MERCs. Although several genetically encoded fluorescent probes for detecting MERCs using GFP-complementation methods have been reported,^103,105,106)^ artificially induced MERCs caused by the expression of these probes need to be considered in interpreting the results. Careful validation of the existing probes and development of probes with fewer artifacts will pave the way for further studies of MERCs in neurons.

## 7 Conclusion

The ER forms an elaborate interconnected network as a center of lipid transport, protein synthesis, and Ca^2+^ regulation throughout all neuronal compartments. In dendrites, previous results imply that the ER locally but dynamically changes its structure to support synaptic plasticity. However, because of the lack of techniques to connect the ultrastructure and temporally dynamic events, it remains unclear how its structural dynamics affect the synaptic plasticity in dendritic spines *in vivo*. Correlative light microscopy-EM analysis can be a powerful tool to address this challenge.^107–110)^ This approach combines time-lapse investigation of neuronal activity by light microscopy, and subsequent imaging of ultrastructural features with EM. In axons, ER remodeling might be important for neurotransmitter release, although where and how the ER structure changes during synaptic transmission and plasticity have not been well described. Furthermore, given that AP-evoked Ca^2+^ response varies between different presynaptic boutons,^111,112)^ the relationship between ER ultrastructure and presynaptic activity in each bouton is of great interest. Further ultrastructural studies at/of presynaptic sites will help to better understand the ER regulation of neurotransmitter release.

Although organelles have been thought of as isolated units, many studies have revealed that they interact with each other. This leads to a major paradigm shift in this field. Although a lot of organelle interactions, especially ER-mitochondria contacts, have been observed in neurons, it remains unanswered how the coordinated actions among organelles contribute to neurotransmission especially in the presynaptic bouton. A clue for answering this question can be provided by studies of ER-membrane contacts in other cell types including yeast. For instance, studies in yeast and mammalian cell lines suggest that the ER-mitochondria contact is important for maintaining mitochondrial DNA that encodes genes essential for the mitochondrial ATP synthesis.^30,113)^ Considering that neurotransmitter release represents a high energy burden,^114)^ it is possible that ER-mitochondria contacts regulate synaptic transmission through the regulation of metabolic homeostasis. Although this review has focused on ER-mitochondria contacts, the ER forms extensive contacts with the plasma membrane (ER-plasma membrane contacts) in mature neurons. Recent reports have revealed that ER-resident proteins VAPs and TMEM24 and plasma membrane localized Kv channels are enriched in these contacts,^115–117)^ whereas the roles of the contact in neurotransmission remain largely unaddressed. Thus, studies of ER-membrane contacts are still in their infancy and will give us new insights in the field of neurotransmission.
